# ADD-ASPIRIN: A phase III, double-blind, placebo controlled, randomised trial assessing the effects of aspirin on disease recurrence and survival after primary therapy in common non-metastatic solid tumours

**DOI:** 10.1016/j.cct.2016.10.004

**Published:** 2016-11

**Authors:** Christopher Coyle, Fay H. Cafferty, Samuel Rowley, Mairead MacKenzie, Lindy Berkman, Sudeep Gupta, C S Pramesh, Duncan Gilbert, Howard Kynaston, David Cameron, Richard H. Wilson, Alistair Ring, Ruth E. Langley, Add-Aspirin investigators

**Affiliations:** a MRC Clinical Trials Unit, UCL, Aviation House, 125 Kingsway, London WC2B 6NH, UK; b Independent Cancer Patient Voices, 17 Woodbridge Street, London EC1R 0LL, UK; c NCRI Consumer Forum, Angel Building, 407 St John Street, London EC1V 4AD, UK; d Room No. 1109, 11th Floor, Homi Bhabha Block, Tata Memorial Centre/Hospital, Parel, Mumbai 400012, India; e Department of Surgical Oncology, Tata Memorial Centre, Dr Ernest Borges Marg, Parel, Mumbai 400012, India; f Sussex Cancer Centre, Royal Sussex County Hospital, Eastern Road, Brighton, Sussex BN2 5BE, UK; g Room 2F65, Block A2, Cardiff School of Medicine, Heath Park, Cardiff CF14 4XN, UK; h Edinburgh Cancer Research Centre, University of Edinburgh, Western General Hospital, Crewe Road South, EH4 2XR, UK; i Centre for Cancer Research and Cell Biology, Queen's University Belfast, 97 Lisburn Road, Belfast BT9 7AE, Northern Ireland, UK; j The Royal Marsden NHS Foundation Trust, Downs Road, Sutton, Surrey SM2 5PT, UK

**Keywords:** Aspirin, Breast cancer, Colorectal cancer, Gastro-oesophageal cancer, Prostate cancer, Randomised controlled trial

## Abstract

**Background:**

There is a considerable body of pre-clinical, epidemiological and randomised data to support the hypothesis that aspirin has the potential to be an effective adjuvant cancer therapy.

**Methods:**

Add-Aspirin is a phase III, multi-centre, double-blind, placebo-controlled randomised trial with four parallel cohorts. Patients who have undergone potentially curative treatment for breast (*n* = 3100), colorectal (*n* = 2600), gastro-oesophageal (*n* = 2100) or prostate cancer (*n* = 2120) are registered into four tumour specific cohorts. All cohorts recruit in the United Kingdom, with the breast and gastro-oesophageal cohort also recruiting in India. Eligible participants first undertake an active run-in period where 100 mg aspirin is taken daily for approximately eight weeks. Participants who are able to adhere and tolerate aspirin then undergo a double-blind randomisation and are allocated in a 1:1:1 ratio to either 100 mg aspirin, 300 mg aspirin or a matched placebo to be taken daily for at least five years. Those participants ≥ 75 years old are only randomised to 100 mg aspirin or placebo due to increased toxicity risk.

**Results:**

The primary outcome measures are invasive disease-free survival for the breast cohort, disease-free survival for the colorectal cohort, overall survival for the gastro-oesophageal cohort, and biochemical recurrence-free survival for the prostate cohort, with a co-primary outcome of overall survival across all cohorts. Secondary outcomes include adherence, toxicity including serious haemorrhage, cardiovascular events and some cohort specific measures.

**Conclusions:**

The Add-Aspirin trial investigates whether regular aspirin use after standard therapy prevents recurrence and prolongs survival in participants with four non-metastatic common solid tumours.

## 1 Background and introduction

### 1.1 Rationale

The Add-Aspirin trial includes participants with breast, colorectal, gastro-oesophageal and prostate tumours which, together, accounts for approximately one third of all cancer cases and cancer deaths [1]. The selected disease sites are those for which (i) the evidence relating to potential benefit of aspirin is strongest; (ii) the potential impact is large (common cancers with large numbers of cases diagnosed at an early stage, or where outcomes of curative treatment are particularly poor); and (iii) recruitment is feasible. As a low-cost pharmaceutical, feasible to administer in both resource poor and rich countries, aspirin has the potential to significantly impact on cancer outcomes worldwide. This, combined with other possible health benefits (such as cardiovascular effects), means that aspirin warrants further investigation as an anti-cancer agent.

### 1.2 Supporting evidence

There have been well over 100 case-control and cohort studies investigating the use of aspirin and cancer risk [2]. A meta-analysis of such studies showed that aspirin use resulted in significant reductions in the risk of developing cancer, most notably in colorectal (relative risk (RR) 0.73, 95% confidence-interval (CI) 0.67–0.79), gastric (RR 0.67, CI 0.54–0.83), adenocarcinoma of the oesophagus/cardia (RR 0.67, CI 0.54–0.83), squamous cell carcinoma of the oesophagus (RR 0.64, CI 0.52–0.78), breast (RR 0.90, CI 0.85–0.95), and prostate cancer (RR 0.90, CI 0.85–0.98) [2]. Observational studies have also shown improvements in survival with aspirin use after a diagnosis of breast [3], [4], [5], colorectal [6], [7], [8], [9], [10], [11], gastro-oesophageal [12], [13] and prostate cancer [14], [15], [16].

Randomised data is available to indirectly substantiate these observations. A meta-analysis of individual participant data on cancer incidence in randomised trials (designed to investigate the effect of aspirin on vascular disease) show marked reductions in cancer incidence and cancer mortality associated with regular aspirin use (greater than three years) in both the short and long-term [17], [18], [19], [20]. Similarly, long-term follow-up from the Women's Health Study, a randomised placebo-controlled trial designed to assess the effects of aspirin (100 mg on alternate days) in the primary prevention of cardiovascular disease and cancer, showed that allocation to aspirin reduced the incidence of colorectal cancer with ten years of follow-up (hazard ratio (HR) 0.80, CI 0.67–0.97) [21]. A recent meta-analysis of studies examining aspirin use after a cancer diagnosis has shown a significant reduction in cancer-specific mortality in colon cancer, but not in breast and prostate cancer, however significant heterogeneity between studies was identified [22].

Furthermore, the first randomised trial specifically designed to demonstrate that aspirin can prevent the development of cancer has shown that 600 mg of aspirin daily for up to four years prevents colorectal and other cancers associated with Lynch syndrome (a hereditary condition which predisposes to cancer development) HR 0.45, CI 0.26–0.79 [23].

The potential benefits of aspirin have to be weighed against the risk of adverse effects. A number of systematic reviews and meta-analyses have examined the potential risks of adverse events [24], [25]. A recent review estimates that, depending on age and sex, regular aspirin use over a 15-year period would lead to an absolute increase in major bleeding events of between 0.16% and 0.81%. The authors conclude that prophylactic aspirin use for a minimum of five years at a dose of 75 mg–325 mg daily has a favourable benefit-harm profile [26]. For individuals treated for cancer, with a high risk of recurrent disease, the balance could be even more favourable.

## 2 Methods

### 2.1 Aims

The Add-Aspirin trial aims to assess whether regular aspirin use after standard potentially curative primary therapy can prevent recurrence and prolong survival in individuals with four common early stage solid tumours. Avoiding recurrent disease, subsequent treatment and the associated morbidity and mortality in these individuals is an important goal. Multicentre and international recruitment will allow assessment of the intervention in a range of settings, with the aim of demonstrating that implementation is both feasible and cost-effective across varying health care systems and in both the developing and developed world. A secondary aim is to assess the potential overall health benefits of aspirin for these individuals including cardiovascular outcomes.

### 2.2 Overview of design

**Fig. 1:**
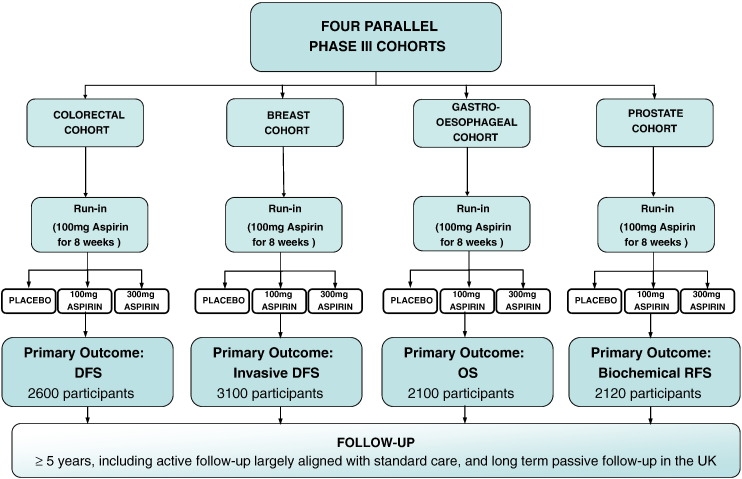
Add-Aspirin trial schema. DFS = Disease free survival, OS = overall survival, RFS = recurrence free survival.

The Add-Aspirin trial investigates the use of both 100 mg daily and 300 mg daily aspirin compared with matched placebo (double-blind) in each of four different tumour types, utilising an overarching protocol. Further details of the rationale for this design are provided in the discussion section of this article. Fig. 1 shows a summary schema for the trial.

### 2.3 Participants

**Fig. 2:**
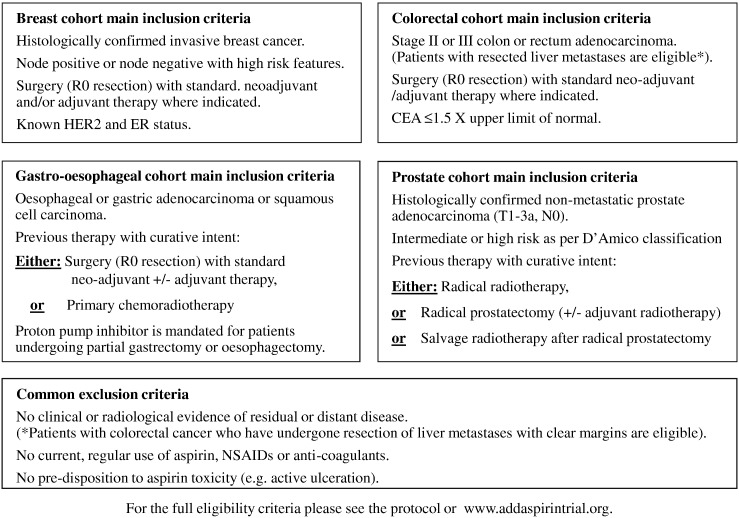
Summary of eligibility criteria.

Participants entering the Add-Aspirin trial have undergone potentially curative treatment (surgery or other radical treatment) for breast, colorectal, gastro-oesophageal or prostate cancer with standard neoadjuvant and/or adjuvant therapy if indicated, and may also have participated in any pre-approved trials and satisfy the eligibility criteria, summarised in Fig. 2.

### 2.4 Registration

The Add-Aspirin trial is open to centres in every Cancer Research Network (CRN) throughout the four devolved nations of the United Kingdom (UK) and will also recruit participants in India (other countries may join subsequently). Eligible participants who have provided consent and meet the timing of entry criteria are registered online (through the trial website, www.addaspirintrial.org). The timing of entry window has been designed so that aspirin can be started at the earliest opportunity to maximise the potential benefits, whilst starting at a time when it is considered safe to do so and unlikely to compromise the curative intent of standard primary treatment. Figures describing the timing of entry criteria for each cohort are available in Appendix B.

### 2.5 Run-in period

The Add-Aspirin trial incorporates a feasibility phase lasting approximately 2 1/2 years during which recruitment feasibility, treatment adherence and safety will be assessed. During the feasibility phase of the study, all participants are required to complete an active run-in period after registration but prior to randomisation where they take 100 mg aspirin daily (one tablet per day) in an open-label manner for a period of approximately eight weeks.

### 2.6 End of run-in period assessment

At the end of the run-in period, the participant's tolerance of aspirin and adherence to daily treatment will be assessed. This approach allows those individuals who are unlikely to be able to tolerate aspirin, as well as those who are unlikely to be able to adhere to the protocol treatment schedule, to be identified. Adherence will be assessed using a combination of a participant diary card, used blister packs and patient reported adherence. Participants will be suitable for randomisation if they have taken at least 80% of their run-in treatment and have not experienced any aspirin-related severe toxicity (defined as ≥ grade 3 CTCAE v4), nor any grade of gastrointestinal bleeding, active gastrointestinal ulceration, new or worsening tinnitus, macular degeneration, intracranial bleeding or hypersensitivity to aspirin. If the investigator feels that the reason for inadequate adherence is temporary (for example, due to toxicity resulting from concomitant adjuvant treatment which has subsequently finished or a non-recurrent unrelated event), the run-in period may be extended by four or eight weeks to reassess adherence and toxicity subject to agreement from the central trial team. Those participants identified as suitable for further study participation, and who remain eligible and are willing to continue in the trial then re-confirm their consent to participate before being randomised.

### 2.7 Randomisation

Following assessment at the end of the run-in period, eligible participants in the UK are randomised by phone and, in India, via the trial website. Participants undergo a double-blind randomisation. Randomisation is performed separately within each tumour-specific cohort and uses minimization algorithms based on key prognostic factors (dependent on tumour site), incorporating a random element. Within each tumour-specific cohort, participants who are below 75 years old are allocated in a 1:1:1 ratio to either 100 mg aspirin, 300 mg aspirin or a matched placebo. Participants who are 75 years old or over, are only allocated to either 100 mg aspirin or matched placebo as toxicity is thought to increase with age but the allocation ratio of 2:1 remains so that they have the same chance of receiving active treatment as the other participants. The target randomisation figure is 9920 participants in the United Kingdom (UK) and India combined. Assuming that approximately 10% of participants will not be randomised following the run-in (for reasons relating either to toxicity or adherence), it is expected that 11,000 participants will be registered to begin the run-in period.

### 2.8 Follow-up

Patients are followed up at three-monthly intervals initially and then six-monthly. Adherence to treatment is verbally assessed at every follow-up visit. In the UK, trial treatment, and active follow-up, continues for at least five years after randomisation. Long-term passive follow-up data will be obtained from routinely-collected healthcare databases for at least ten further years. Indian participants will be actively followed-up for at least ten years after randomisation. For participants that are registered but do not go on to be randomised, active participation in the trial will end at that time. However, passive follow-up will continue via routinely-collected healthcare datasets where consent for this has been obtained. The trial assessment schedule for each cohort is aligned with standard practice where possible to ensure they can be implemented easily. This is balanced with the need to ensure appropriate monitoring of patients on trial treatment and assessment of outcome measures. The trial follow-up schedules are available in Appendix C.

### 2.9 Toxicity management

Participants that experience any aspirin-related severe toxicity (defined as ≥ grade 3 Common Terminology Criteria for Adverse Events (CTCAE v4)) or any grade of gastrointestinal bleeding, active gastrointestinal ulceration, tinnitus, macular degeneration, intracranial bleeding or hypersensitivity to aspirin are required to permanently discontinue aspirin immediately.

For those who are asymptomatic, prophylactic measures to reduce the risk of gastrointestinal toxicity from aspirin (such as proton pump inhibitor (PPI) prophylaxis and helicobacter pylori eradication) are not routinely recommended in participants at low risk of gastrointestinal complications and so are not mandated in the Add-Aspirin trial protocol. However, PPI use for the duration of aspirin treatment is recommended for patients who have undergone oesophagectomy or partial gastrectomy and should also be considered for older patients (≥ 75 years), or any other participant who might be at increased risk of toxicity. Intracranial bleeding is a rare toxicity of aspirin, and hypertension can increase the risk. Those with poorly controlled hypertension have trial treatment withheld until their blood pressure is controlled. Further guidelines are available in the trial protocol.

Investigators are advised to manage toxicities under the assumption that the participant is receiving the highest possible dose of the active product (300 mg aspirin), without the need for unblinding, however where knowledge of treatment allocation would alter clinical management, unblinding is possible. Unblinding can be performed via an access-controlled system available through the trial website (www.addaspirintrial.org).

### 2.10 Sub-studies

The size and diversity of the Add-Aspirin cohort provides opportunity to address other secondary research questions and evaluate novel methodology. Aspirin has been proposed to have a number of health benefits beyond cancer, particularly in older people. To investigate the overall health benefits of aspirin, functional capacity is assessed using the Vulnerable Elders Survey (VES-13) [27]. This is performed for participants that are 65 years old or over at trial registration, and five years after randomisation and can be administered in person or over the telephone. The hypothesis that aspirin protects against cognitive decline is assessed using a short version of the Montreal Cognitive Assessment (the MoCA-blind). The MoCA-blind takes approximately 7 to 10 min to complete and is administered in all Add-Aspirin trial participants at registration, then again at one and five years after randomisation. No training is required to administer the questionnaire, which can be conducted in person, or over the telephone. A methodological sub-study will compare the quality and completeness of routinely-collected healthcare data with data collected within the trial, with the aim of assessing the suitability of passive follow-up data collection for investigating long-term primary and secondary outcome measures within the trial. This will be an early validation that will determine ongoing use of routinely collected data in the trial. A sub-study is planned to investigate methods of measuring adherence, including the collection of urine samples to measure thromboxane B2 (a direct measure of the effects of aspirin). Methodological sub-studies to improve site initiation and recruitment will also be undertaken.

### 2.11 Translational objectives

The Add-Aspirin trial incorporates a sample repository where a baseline blood sample and tumour sample are stored for future translational projects. The sample repository is jointly hosted by two institutions in the UK, Tayside Tissue Bank and the Wales Cancer Bank. In India, a baseline blood sample and tumour sample from selected sites will be stored at the Tata Memorial Centre biobank. A number of studies are expected to be initiated whilst the trial is ongoing (subject to funding), including studies to identify groups that will benefit most from aspirin use (for example, investigation of the role of tumour PIK3CA mutation status), and to investigate the mechanisms underlying the anti-cancer effects of aspirin, particularly effects on platelet function and the pro-thrombotic tumour microenvironment.

## 3 Results

### 3.1 Outcomes

Tumour site-specific primary analyses will take place 5–6 years after recruitment of the last participant for that cohort, with the exact timing based on the observed numbers of events. Primary and secondary outcome measures are available in Table 1. Overall survival is a secondary outcome measure in all cohorts except the gastro-oesophageal cohort, where it is the primary outcome. Overall survival will also be assessed as a co-primary outcome measure in all participants after 15 years. The longer follow-up and large sample size associated with this analysis will enable any long-term benefits of aspirin to be realised, including those unrelated to the primary cancer, for example the potential for prevention of deaths related to vascular events and second malignancies. Consideration of rates of serious toxicity (and particularly serious haemorrhage), as well as other secondary health outcomes, alongside the efficacy results will be particularly important in these analyses in order to provide an holistic assessment of the potential risks and benefits associated with different doses.

### 3.2 Statistical considerations

Primary analyses will compare outcomes for participants allocated to aspirin (100 mg and 300 mg arms combined) and participants allocated to placebo, regardless of the treatment received (i.e. intention-to-treat). The primary analyses will include both those participants < 75 years who underwent the full randomisation and those ≥ 75 years who underwent randomisation between 100 mg aspirin or placebo only, but the dose effects of aspirin will be investigated only on those randomised between the two doses.

If an overall effect of aspirin vs. placebo is observed in the primary treatment comparison for one or more cohorts, a further analysis will be performed to investigate differences in efficacy according to aspirin dose. This analysis will be performed only in the cohorts that show a positive result for aspirin vs. placebo and will be stratified by cohort. By making these analyses conditional on a benefit of aspirin being observed in the primary analysis, the likelihood of a false-positive result is reduced. The rationale for combining the data across cohorts is to maximise power, as we anticipate that any difference between doses of aspirin will be smaller than the difference between aspirin and placebo. The trial has also established collaborative links with research groups running other aspirin cancer trials internationally with a view to future meta-analyses.

### 3.3 Sample size breast cohort

Based on data from recent trials, we expect that five-year invasive disease-free survival (IDFS) in the control group will be approximately 80% [29], [30], [31], [32]. 717 IDFS events will be required to achieve 90% power to detect a 4% (HR = 0.78) improvement in this rate. Assuming that the cohort takes 3 1/2 years to recruit, with analysis six years later, we anticipate that 3100 participants will be required to observe this number of events.

### 3.4 Sample size colorectal cohort

Based on data from recent trials, we expect that five-year disease-free survival (DFS) in this cohort will be approximately 70% [33], [34]. 899 DFS events will be required to achieve 90% power to detect a 5% (HR = 0.80) improvement in this rate. Assuming that the cohort takes 3 1/2 years to recruit, with analysis six years later, we anticipate that 2600 participants will be required to observe this number of events.

### 3.5 Sample size gastro-oesophageal cohort

Based on data from recent trials, we expect that five-year overall survival in this cohort will be approximately 45% [35], [36], [37], [38], [39]. 1120 deaths will be required to achieve 80% power to detect a 6% (HR = 0.84) improvement in this rate. Assuming that the cohort takes six years to recruit, with analysis five years later, we anticipate that 2100 participants will be required to observe this number of events.

### 3.6 Sample size prostate cohort

The radical prostatectomy and radical radiotherapy groups are powered to assess effects separately. In the radical prostatectomy group, we anticipate that biochemical recurrence-free survival (bRFS) at five years will be approximately 75% [40]. For the radical radiotherapy group, five year bRFS is estimated to be approximately 65% [41]. To achieve 90% power to detect an 8% improvement in these rates, 673 bRFS events will be required. Assuming that the cohort takes five years to recruit, with analysis five years later, we anticipate that 2120 participants will be required to observe this number of events.

Sample size calculations for all cohorts are based on a two-sided 5% significance level and account for a degree of loss to follow-up and slower recruitment in the early stages of the trial. Target registrations have been inflated to allow a 10% dropout after the run-in period.

### 3.7 Ethical considerations

The trial will be conducted in compliance with the approved protocol, the Declaration of Helsinki 2008, the principles of Good Clinical Practice (GCP), Commission Directive 2005/28/EC with the implementation in national legislation in the UK by Statutory Instrument 2004/1031 and subsequent amendments, the UK Data Protection Act (DPA number: Z5886415), and the National Health Service (NHS) Research Governance Framework for Health and Social Care (RGF). International centres will comply with the principles of GCP as laid down by the ICH topic E6 (Note for Guidance on GCP) and applicable national regulations. The Add-Aspirin trial is registered with the International Standard Randomised Controlled Trial Number ISRCTN74358648, and has also been submitted for registration with the Clinical trials Registry of India (REF/2016/06/011465). The Add-Aspirin trial was approved by the South Central – Oxford C research ethics committee and is part of the UK National Cancer Research Network (NCRN) portfolio. In India, the trial has been approved by the Directorate of the National Cancer Grid (NCG), and is part of the NCG trials portfolio. University College London (UCL) and the Tata Memorial Centre (TMC) are co-sponsors of the trial and have delegated responsibility for the overall management of the Add-Aspirin trial to the MRC CTU at UCL and Tata Memorial Centre CTU for India.

## 4 Discussion

The Clinical Trials Authorisation for the Add-Aspirin trial was granted by the Medicines and Healthcare products Regulatory Agency (MHRA) on 25th November 2014, and ethics approval was given on 4th June 2014. The first participant was recruited on 8th October 2015. At the time of writing on 1st May 2016, the Add-Aspirin trial is recruiting across 120 centres in the UK, and is recruiting ahead of its overall projected targets, with the breast cohort the fastest recruiter. The last of the four cohorts is now expected to complete recruitment in 2021. Current recruitment figures are available at www.addaspirintrial.org.

### 4.1 Challenges and methodological solutions

There are a number of practical and operational challenges presented by a large adjuvant trial of a generic and repurposed intervention. These include the need for cost efficiencies due to a lack of industry financial support for a trial of a generic pharmaceutical; ensuring sufficient long-term adherence in a largely asymptomatic population; and the potential for control arm contamination due to over the counter (OTC) availability of aspirin. These have been addressed using a number of contemporary methodological approaches.

### 4.2 Overarching protocol

A platform trial design was chosen because there is evidence that aspirin is potentially effective in multiple tumours. Investigating the use of both 100 mg daily and 300 mg daily aspirin across cohorts addresses uncertainty surrounding the optimal aspirin dose required to achieve anti-cancer effects, potentially saving many years of research time. Keeping all four cohorts within a single protocol ensures that the management of each cohort is as comparable as possible (with the exception of some site specific procedures). This allows a combined analysis of overall survival as a co-primary outcome measure and cross cohort secondary analyses of toxicity, cardiovascular and other health benefits, thus increasing the overall potential impact of the trial. In addition, this design provides the capacity to add further tumour sites and also provides a potential platform for evaluation of other repurposed agents. An overarching protocol provides economies of scale both centrally and site level, including site set-up, regulatory approval, central staffing, coordination, oversight and data management. The resulting cost efficiencies improve the financial viability of the trial given the lack of industry support, and provide value for money for our charitable and governmental funders.

### 4.3 Antecedent aspirin use

A proportion of individuals who are otherwise eligible for the trial will already be taking aspirin regularly. It is conceivable that pre-existing aspirin use could alter tumour biology [42], and that those already taking aspirin might be randomised to placebo which would be unethical. Consequently a decision was made to exclude current or previous regular aspirin users.

### 4.4 Over the counter aspirin

Unlike most other cancer trials, the intervention in the Add-Aspirin can be purchased without a prescription. There is a risk that some potential participants might opt to not enter the trial and purchase aspirin independently. OTC availability of aspirin also leads to the potential for control arm contamination and an increased risk of toxicity if they are randomised to 300 mg aspirin. To combat OTC aspirin use, site staff are trained to re-enforce the key message that as yet, there is no clear evidence from a randomised trial that adjuvant aspirin use improves survival, and equipoise is emphasised in all information provided to potential participants. For those already registered, site staff are trained to regularly ask about and discourage OTC aspirin use. Randomisation to two doses of aspirin or placebo also leads to a 2:1 chance of receiving a potentially active agent.

### 4.5 Timing of entry considerations

The timing of trial registration has been aligned across all four cohorts to make the treatment of each as similar as possible, however a number of adjustments have been necessary to account for the variety of treatment modalities and pathways, and differences in patient characteristics between cohorts. The timing of entry around adjuvant chemotherapy requires particular consideration. The risk of developing dyspepsia during adjuvant chemotherapy varies according to the regimen used, the need for dexamethasone as a supportive therapy, and the incidence of risk factors in that group. Dyspepsia is a common occurrence during adjuvant chemotherapy for breast cancer and as such, patients can only register once chemotherapy is complete. In the colorectal and gastro-oesophageal cohorts dyspepsia developing as a consequence of adjuvant chemotherapy is less common, and registration is permitted when six weeks of adjuvant chemotherapy has been administered (without developing dyspepsia and subject to acceptable platelet counts).

### 4.6 Run-in period

The run-in period was implemented after funders' concern about the risk of poor adherence. Adherence will be assessed during the run-in period using three different methods to allow a more accurate assessment. These include a participant diary card, return of used blister packs and participant interview at an end of run-in assessment. The run-in period provides an early opportunity to assess feasibility, in terms of early toxicity, recruitment and patient acceptability, and provides a population for the randomised phase who are more likely to tolerate and adhere to the trial treatment for the duration of the trial [43]. This strategy has also been used successfully in other aspirin trials [44], [45]. We do not believe that eight weeks of aspirin will reduce the effect between the aspirin and placebo comparison in the trial as data have consistently shown that long-term treatment (a minimum of 2 years [1], and up to 5–10 years [2], [3]) with aspirin is required for the anti-cancer effects of aspirin to become identifiable [1], [2], [3]. The use of the run-in period is being monitored carefully and will be reviewed on completion of an initial feasibility phase. Other methods of encouraging adherence include the use of blister packs labelled with days of the week, provision of diary cards, participant newsletters and promotion of the trial website which includes updates and reaction to stories related to aspirin in the news media.

### 4.7 Drug supply

Aspirin is a generic drug, Bayer AG donated all doses of the blinded active intervention and matched placebo, but not the packaging, labelling, blinding or distribution, which therefore represented a major operational and funding challenge. These challenges have been met by outsourcing some of these processes and development of an in-house drug supply management system to track stock levels at sites and automatically trigger re-orders based on projected demand. The system also includes an unblinding capability.

### 4.8 Co-enrolment

Since aspirin is intended to be given following or alongside standard primary therapy, rather than replacing any element of current treatment, it will be appropriate to include participants who have already taken part in trials of primary treatments wherever possible. This will allow assessment of the efficacy of aspirin in participants who have received both current and potentially future standard of care treatments. Including participants from other treatment trials will help to ensure the future relevance of both trials, is important for recruitment feasibility, and maximises the opportunities for patients to participate in trials. We have found that the acceptability of co-enrolment to researchers varies by tumour group. This may be due to variation in the amount of trial activity or even differences in patient group demographics. Our approach has been to consider co-enrolment on a trial-by-trial basis, discussing this with the relevant trial teams, with a careful assessment of any conflicts in eligibility criteria, scheduling and potential impact on safety and the results of either trial. Where concerns exist, or reassurance is required, statistical modelling is conducted to assess the potential impact on trial results (there is often limited overlap leading to negligible impact). Co-enrolment has been agreed with 16 trials to-date and is planned with other trials currently in development.

### 4.9 Recruitment in India

Since aspirin is easily available worldwide, demonstrating its implementation in different resource settings will increase the global impact of the results. Recruitment in India is also important to ensure adequate recruitment in the gastro-oesophageal cohort and also allows the development of new international collaborations. Academic multi-centre trials are rare in India and the set-up of Add-Aspirin has helped with development of a research infrastructure for this and future trials. Recent changes in clinical regulations in India have delayed opening and the trial is anticipated to open in India later in 2016.

### 4.10 Conclusions

Aspirin is a low-cost, generic drug that is easily available worldwide. Consequently, if aspirin is shown to be beneficial as an adjuvant treatment, even with a modest effect, it would change practice globally. Compared with many new agents or complex regimens, the intervention could be implemented quickly and on a broad scale, including in lower resource settings, with the potential to have a huge impact on the global cancer burden.

## Funding

The Add-Aspirin trial is jointly funded by Cancer Research UK (CRUK) (grant number C471/A15015) and the National Institute Health Research (NIHR) Health Technology Assessment Programme (HTA) (project number 12/01/38). Bayer Pharmaceuticals AG has agreed to provide the Investigational Medicinal Products (IMPs). In India, the Sir Dorabji Tata Trust provides funding, and CIPLA Ltd. is providing supplies of aspirin 100 mg for the run-in period. The Add-Aspirin translational sample collection is funded by CRUK (C471/A19252). The trial is coordinated and supported by the Medical Research Council Clinical Trials Unit at University College London (MRC CTU at UCL).

## Disclosures

Ruth E. Langley has received financial support through grants from Cancer Research UK and the National Institute for Health Research (NIHR) Health Technology Assessment (HTA) as Chief Investigator of the Add-Aspirin trial, has received compensation from Bayer and Aspirin Foundation for service on scientific advisory boards, and has received a supply of aspirin and placebo from Bayer Pharmaceuticals AG for the Add-Aspirin trial. Richard Wilson has received a supply of aspirin and placebo from Bayer Pharmaceuticals AG for the FOCUS4-B trial in metastatic colorectal cancer and for which he is the co-Chief Investigator.

## Figures and Tables

**Table:** 

*Gastro-oesophageal Cohort Trial Management Group*		
Professor Ruth Langley	Chief Investigator and Lead Investigator, Gastro-oesophageal Cohort (UK)	London, UK
Professor C S Pramesh	Lead Investigator – India, Lead Investigator, Gastro-oesophageal Cohort (India)	Mumbai, India
Dr Richard Hubner	Medical Oncologist – Gastro-oesophageal Cohort	Manchester, UK
Professor Janusz Jankowski	Gastroenterologist – Gastro-oesophageal Cohort	Warwick, UK
Mr Tim Underwood	Surgeon – Gastro-oesophageal Cohort	Southampton, UK
Professor Anne Thomas	Medical Oncologist – Gastro-oesophageal Cohort	Leicester, UK
Verity Henson	Clinical Trials Officer	Bristol, UK
Professor John Bridgewater	Medical Oncologist – Gastro-oesophageal Cohort	London, UK

*Breast Cohort Trial Management Group*		
Dr Alistair Ring	Lead Investigator – Breast Cohort (UK)	London, UK
Professor David Cameron	Medical Oncologist – Breast Cohort. Lead Investigator, Translational Research	Edinburgh, UK
Professor Sudeep Gupta	Lead Investigator – Breast Cohort (India)	Mumbai, India

*Colorectal Cohort Trial Management Group*		
Professor Richard Wilson	Lead Investigator – Colorectal Cohort	Belfast, UK
Dr Tim Iveson	Medical Oncologist – Colorectal Cohort	Southampton, UK
Professor Robert Steele	Surgeon – Colorectal Cohort	Dundee, UK
Dr Daniel Swinson	Medical Oncologist – Colorectal Cohort	Leeds, UK
Ms Farhat Din	Surgeon – Colorectal Cohort	Edinburgh, UK

*Prostate Cohort Trial Management Group*		
Professor Howard Kynaston	Lead Investigator – Prostate Cohort	Cardiff, UK
Dr Duncan Gilbert	Clinical Oncologist – Prostate Cohort	Brighton, UK
Mr Paul Cathcart	Surgeon – Prostate Cohort	London, UK

*Cross-Study Collaborators*		
Professor Mahesh Parmar	Director, MRC CTU	London, UK
Professor Peter Rothwell	Clinical Neurologist	Oxford, UK
Professor Carlo Patrono	Pharmacologist	Rome, Italy
Professor Sir John Burn	Clinical Geneticist	Newcastle, UK
Dr David Adlam	Cardiologist	Leicester, UK
Dr Michael Peake	Clinical Lead, National Cancer Intelligence Network	London, UK

*Participant Representatives*		
Lindy Berkman	Participant Representative, NCRI Consumer Forum	UK
Mairead MacKenzie	Participant Representative, Independent Cancer Patient Voices	UK
Vandana Gupta	Participant Representative, VCare	India
Arnold Goldman	Participant Representative	UK

*MRC CTU at UCL*		
Marta Campos	Trial Manager	MRC CTU, UK
Anna Thomason	Trial Manager	MRC CTU, UK
Ben Sydes	Data Manager	MRC CTU, UK
Shabinah Ali	Data Manager	MRC CTU, UK
Alex Robbins	Data Manager	MRC CTU, UK
Sam Rowley	Statistician	MRC CTU, UK
Dr Fay Cafferty	Project Leader/Senior Statistician	MRC CTU, UK
Dr Chris Coyle	Trial Physician and Trial Clinical Research Fellow	MRC CTU, UK

**Figure:**
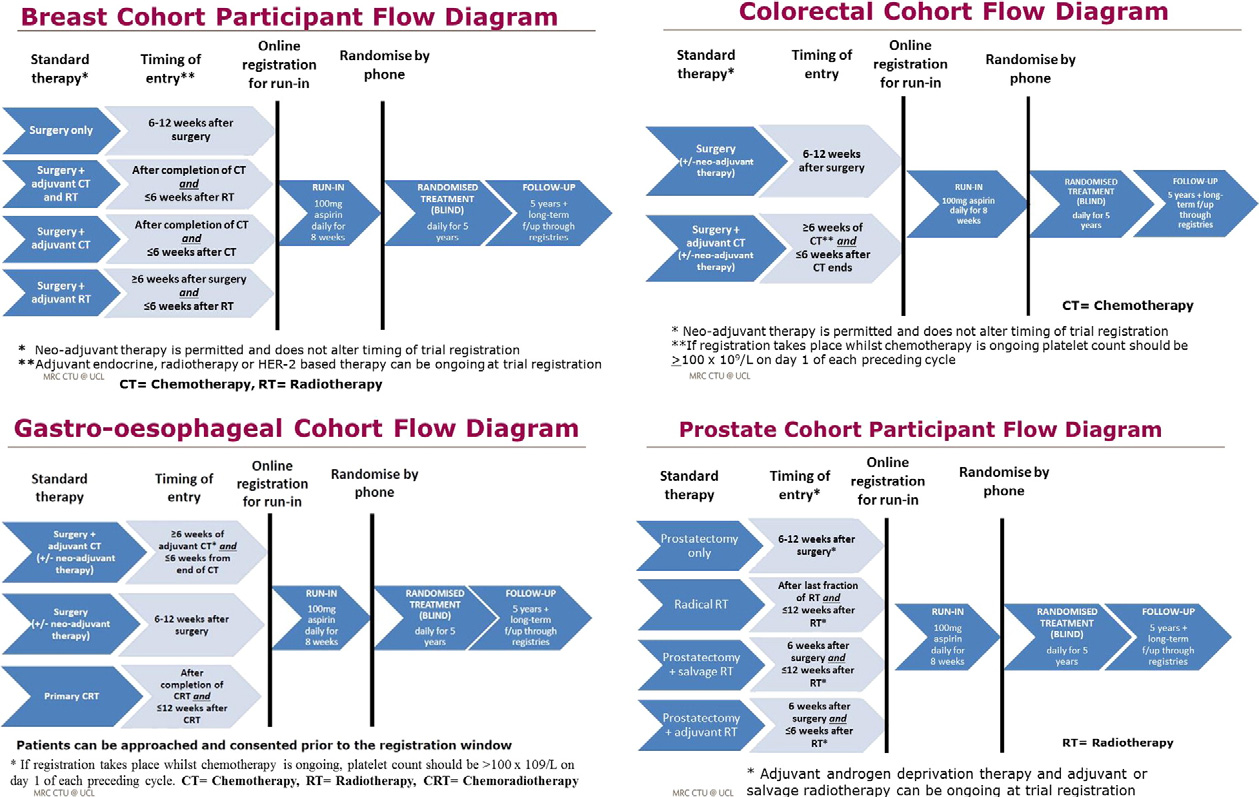


**Table:** 

Assessments		Prior to registration	Prior to randomisation (end of run-in period)	Months since randomisation											
				3	6	9	12	18	24	30	36	42	48	54	60
All cohorts															
Main assessments	Registration assessments^a^	✓													
	End of run-in assessment^b^		✓												
	Follow-up assessments^c^			✓	✓	✓	✓	✓	✓	✓	✓	✓	✓	✓	✓
Intermittent assessments	VES-13 questionnaire (65 ≥ years at registration)	✓													✓
	Cognitive assessment	✓					✓								✓
Blood tests	FBC, LFT, U&E & eGFR	✓	✓		✓		✓		✓		✓		✓		✓
	C-Reactive Protein (CRP)	✓			✓		✓								
	Fasting lipid profile	✓													
Other tests	Tumour and blood sample	✓													
Breast cohort															
Imaging	Mammography	✓					✓		✓		✓		✓		✓
Colorectal cohort															
Imaging and procedures	CT (chest/abdomen/pelvis)	✓					✓		✓						✓
	Colonoscopy	✓						✓		✓					✓
Blood tests	CEA test	✓	✓	✓	✓	✓	✓	✓	✓	✓	✓	✓	✓	✓	✓
Prostate cohort															
Blood tests	PSA	✓	✓	✓	✓	✓	✓	✓	✓	✓	✓	✓	✓	✓	✓
Gastro-oesophageal cohort															
No cohort specific investigations															

a Registration assessments include: eligibility, co-enrolment (if applicable), blood pressure, height, weight, blood results, concomitant medication and comorbidities.

b End of run-in assessments include: symptoms and toxicity, adherence, blood pressure.

c Follow-up assessments include: symptoms and toxicity, adherence, blood pressure, weight, concomitant medication.

**Table 1:** Outcome measures.

Cohort	Primary outcome measures
Breast cancer	Invasive disease-free survival (IDFS) [28]
Colorectal cancer	Disease-free survival (DFS)
Gastro-oesophageal cancer	Overall survival
Prostate cancer	Biochemical recurrence-free survival (bRFS)
All cohorts combined	Overall survival

Cohort	Secondary outcome measures
All cohorts	Overall survival (except for gastro-oesophageal cohort)
	Adherence
	Toxicity
	Serious haemorrhage CTCAE (v4) grade 3 or greater
	Serious vascular events
	Thrombotic events
	Diabetes and associated complications
	Second malignancies
	Age-related macular degeneration
	Cognitive assessment (using the MOCA-blind questionnaire)
	Dementia
	Comorbidities (using the Charlson Index)
	Obesity (using the Body Mass Index)
	Functional capacity (using the VES-13 questionnaire)
Breast	Breast cancer-specific survival
	Bone metastases-free survival
	Invasive disease-free survival-ductal carcinoma insitu (IDFS-DCIS)
Colorectal	Colorectal cancer-specific survival
Gastro-oesophageal	Disease-free survival
Prostate	Prostate cancer-specific survival
	Time to initiation of salvage treatment
	Bone metastases-free survival
